# Supplementary material for: Daily activity rhythms, sleep and pregnancy are fundamentally related in the Pacific beetle mimic cockroach, Diploptera punctata
Source: J Exp Biol. 2025 Aug 4;228(15):jeb250486. doi: 10.1242/jeb.250486 (PMC12377814; doi:10.1242/jeb.250486)
Supplement: Supplementary information [file jexbio-228-250486-s1.pdf]

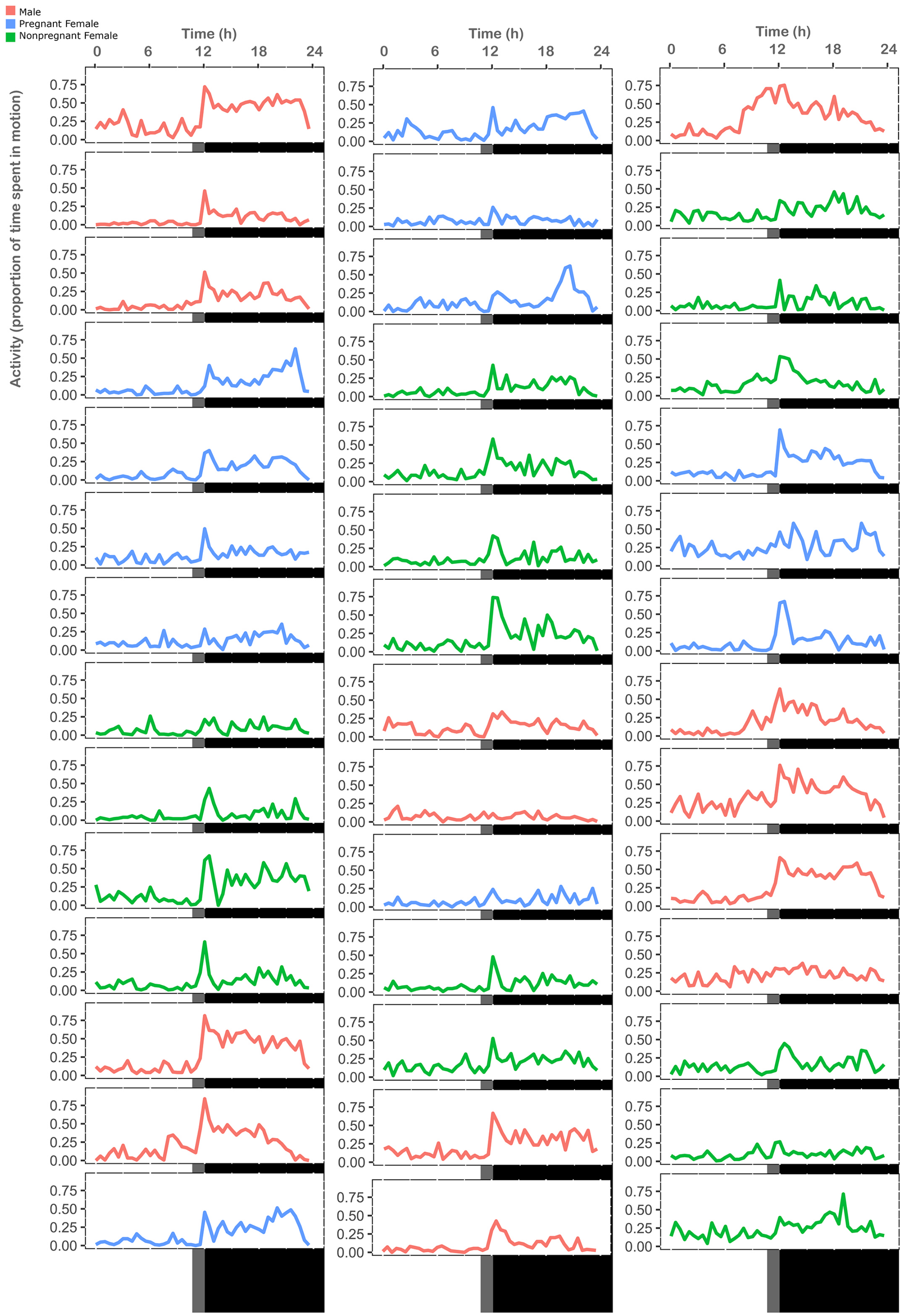
Male
Pregnant Female
Nonpregnant Female
Time (h)
0
6
12
18
24
Activity (proportion of time spent in motion)
0.75
0.50
0.25
0.00

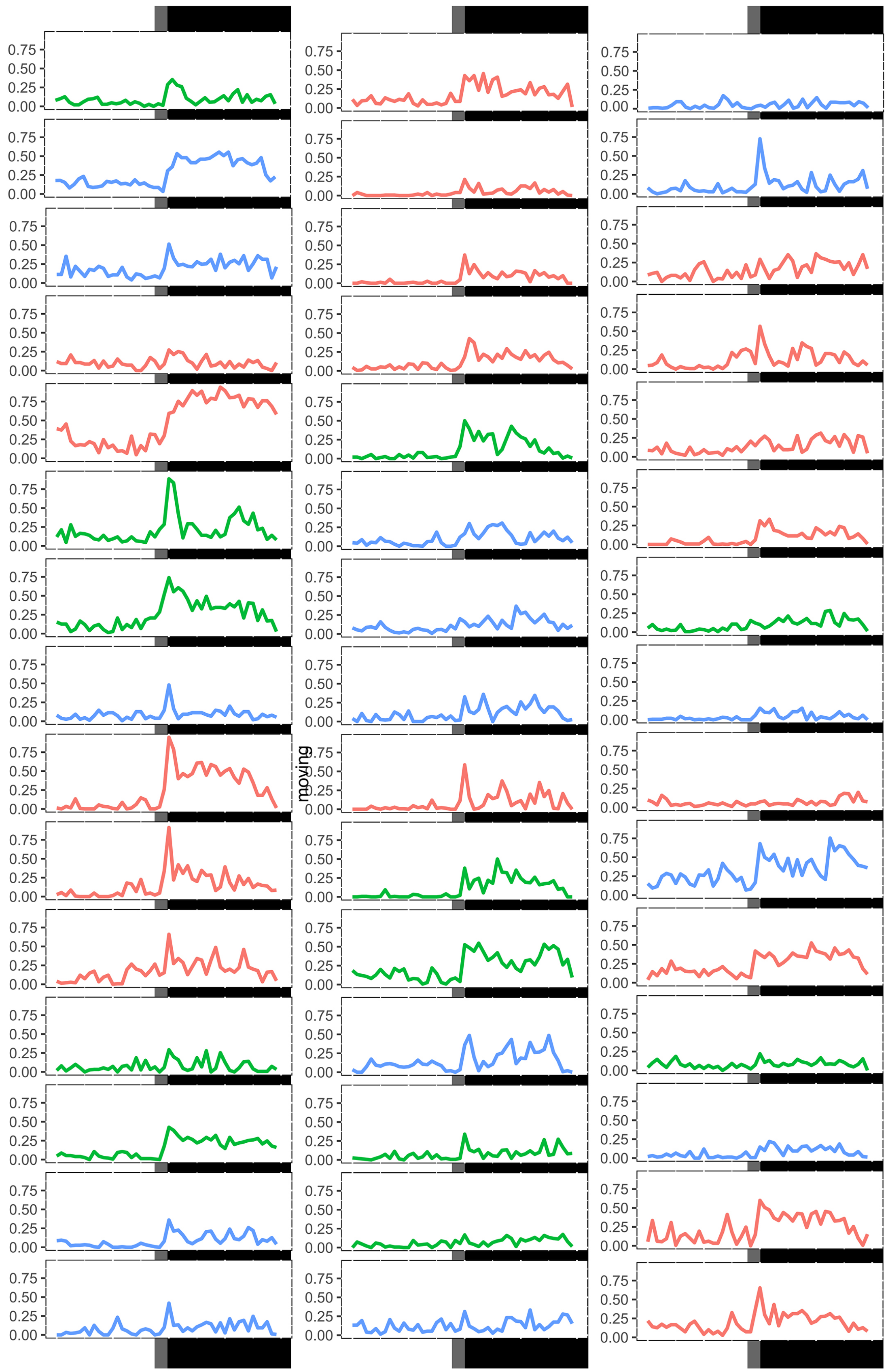
moving
0.75
0.50
0.25
0.00

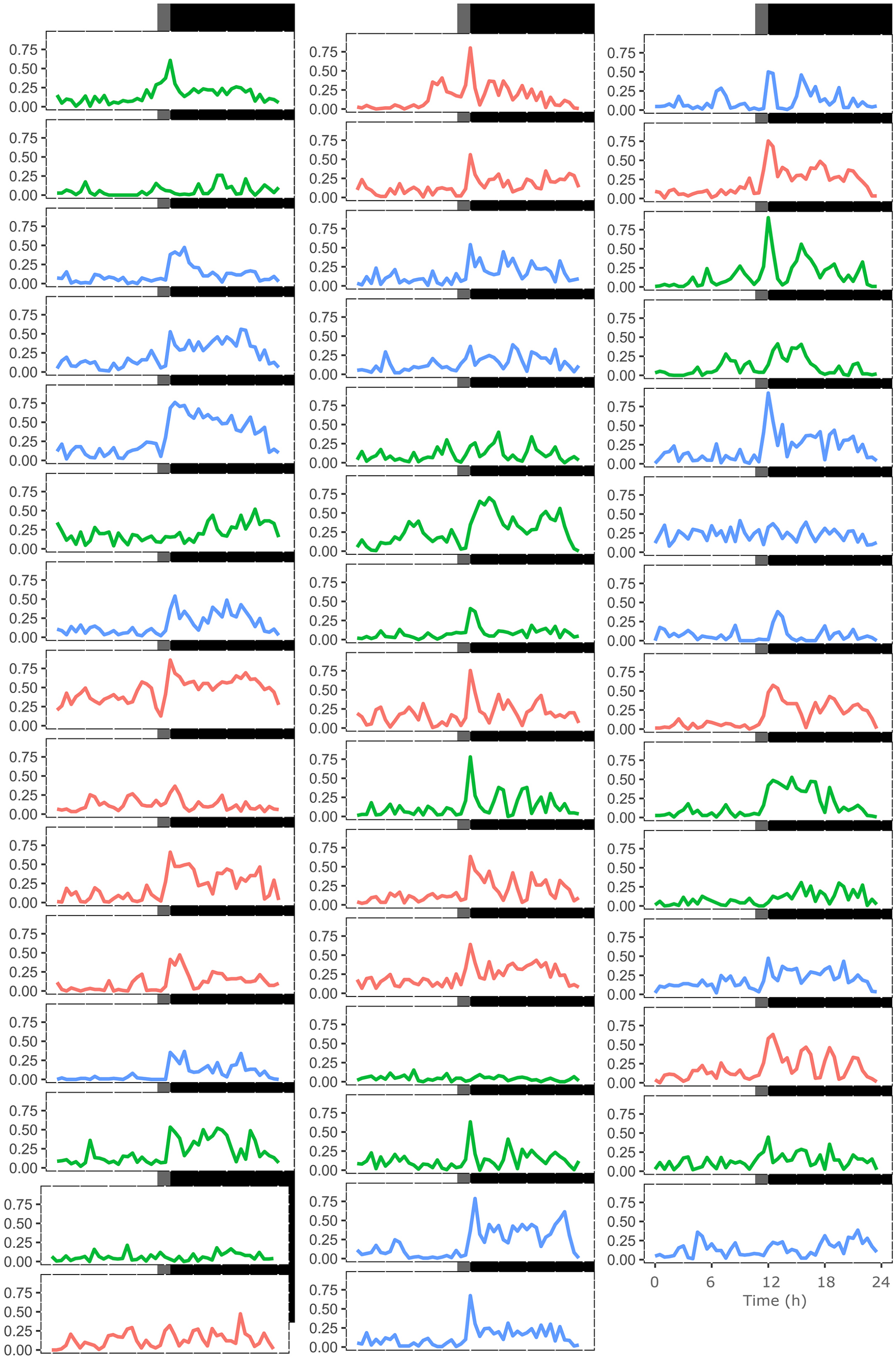

Fig. S1. Activity levels (measured as proportion of time spent moving using single monitor setup) during photophase, dimming, and scotophase by individual. Individuals align with aggregated data, leading us to believe that no one animal is causing the observed trends.

Journal of Experimental Biology • Supplementary information

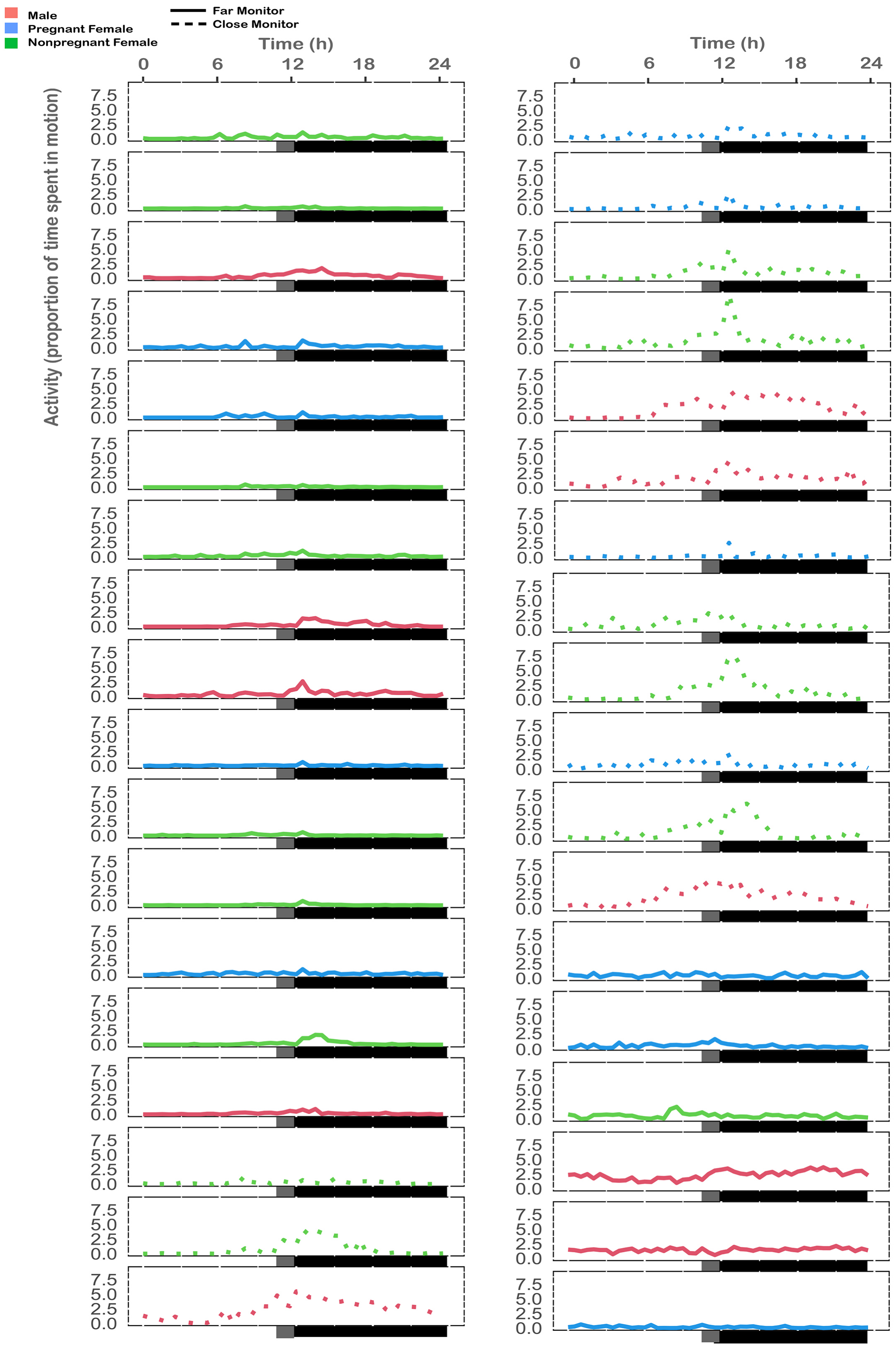

Fig. S2. Activity levels (measured as beam crosses per minute using dual monitor setup) during photophase, dimming, and scotophase by individual. Individuals align with aggregated data, leading us to believe that no one animal is causing the observed trends.

Journal of Experimental Biology • Supplementary information

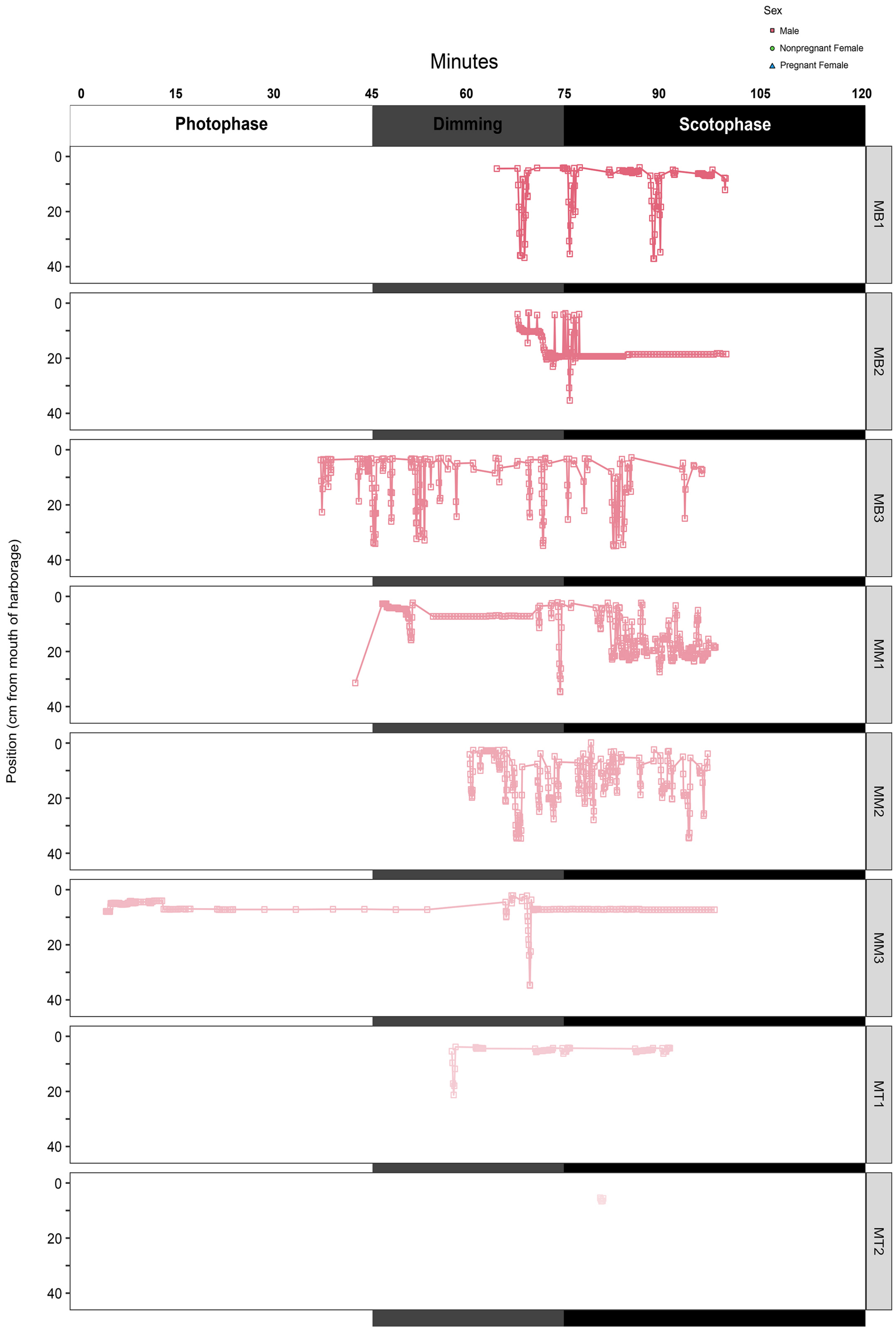

Journal of Experimental Biology • Supplementary information

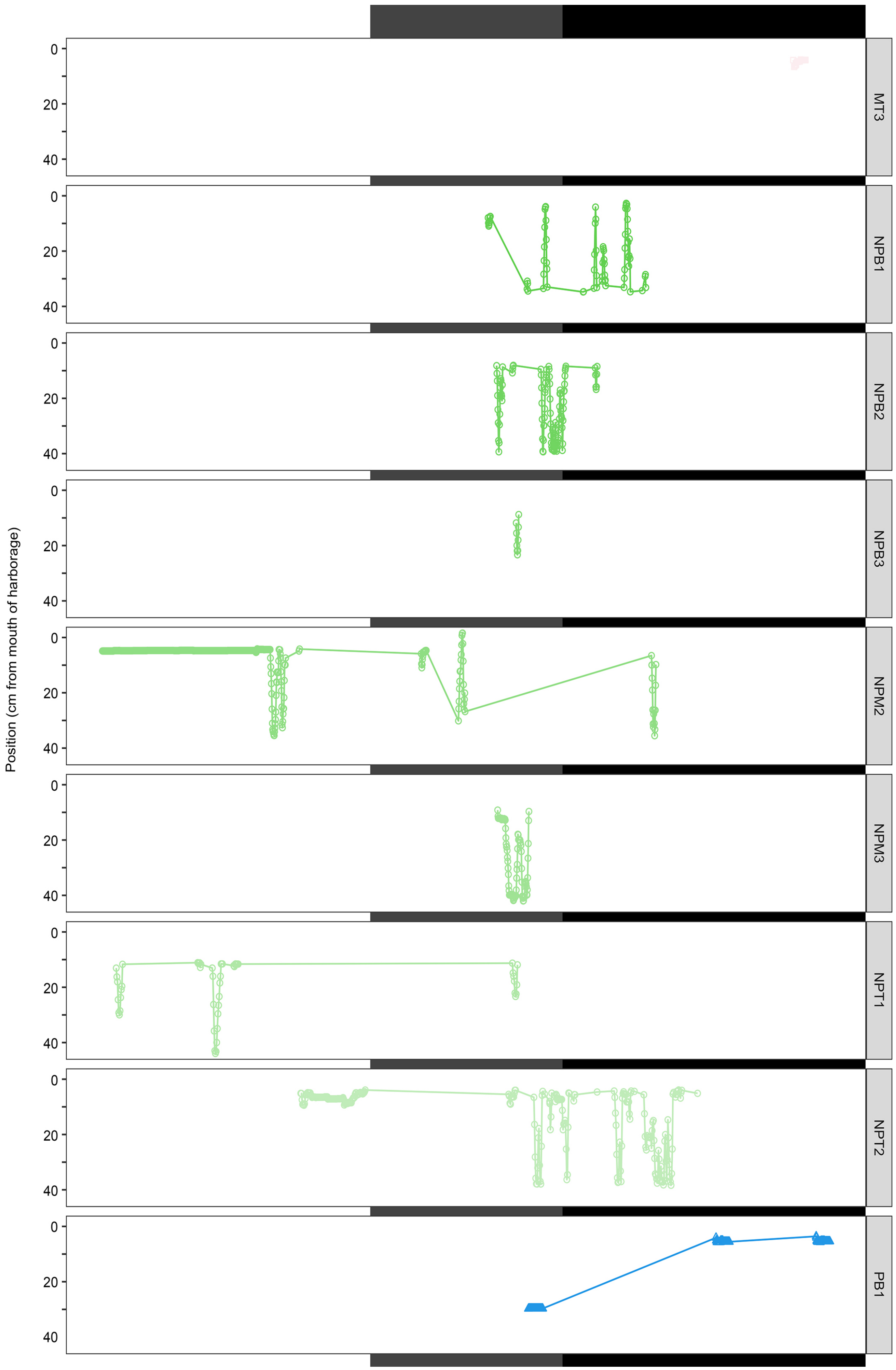

Journal of Experimental Biology • Supplementary information

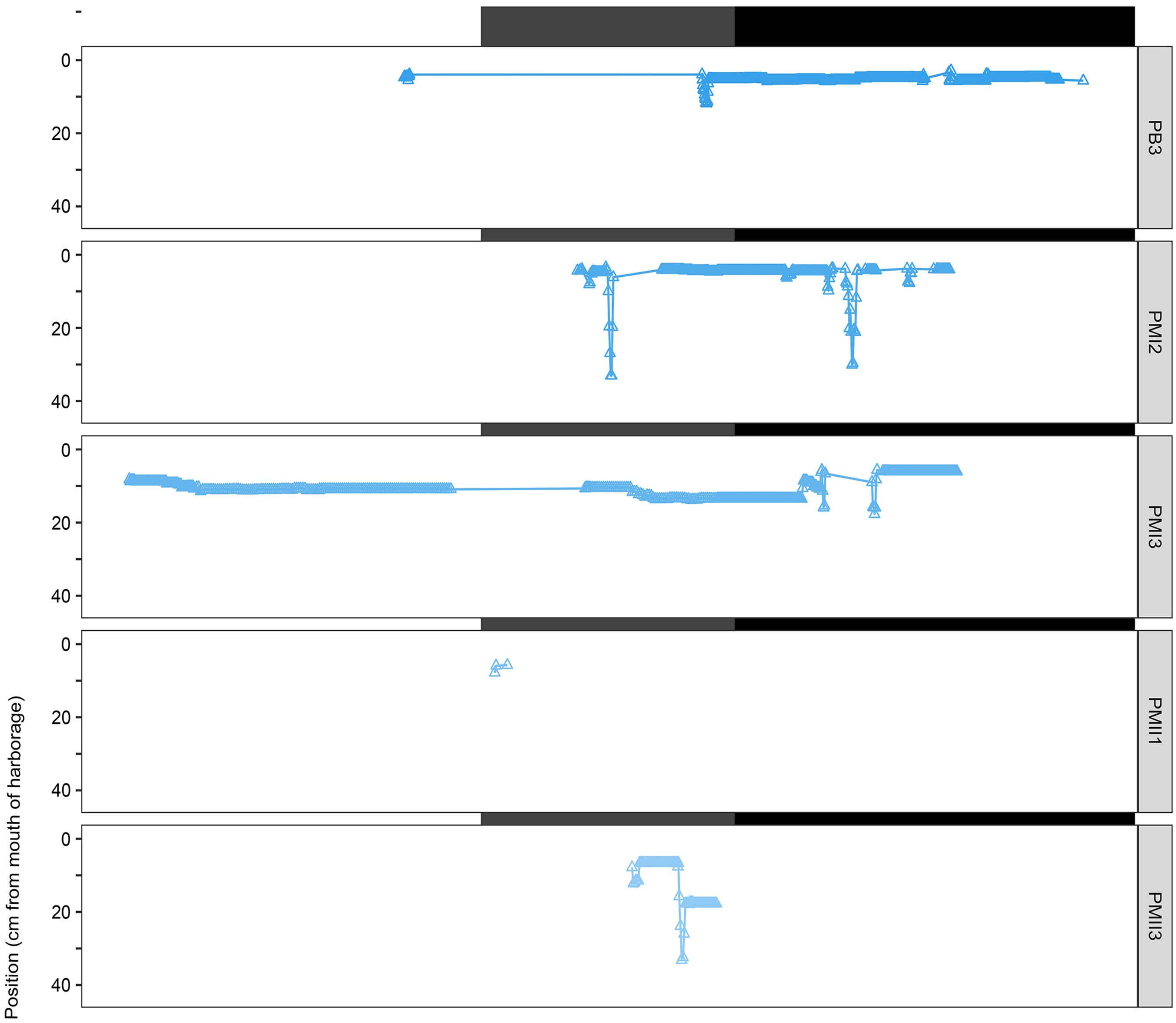

Fig. S3. Position in the tube over time during photophase and scotophase by individual. This two hour period is a portion of the 2.5 hour period described in the dual monitor trials, centered on the dimming period. Individuals align with aggregated data, leading us to believe that no one animal is causing the observed trends.

Journal of Experimental Biology • Supplementary information

Table S1.

**Figure 2 Statistics** All groups *n* = 80	***Variance***	***df***	***P* value**
**Beam Count by Group Kruskal Wallis Test**	x^2 = 2.6687	2	0.2633
**Beam Count by Phase Kruskal Wallis Test**	x^2 = 2.486	2	0.2885
**Day Beam Count by Group Kruskal Wallis Test**	x^2 = 2.3521	2	0.3085
**Night Beam Count by Group One-way ANOVA Test**	F = 1.035<br>Residuals: df 128, sum sq 53.93 , mean sq 0.4213	2	0.358
**Total Sleep Minutes by Group**<br><br>Kruskal-Wallis Test<br><br>Dunn Test<br>Male vs Non-Pregnant<br>Male vs Pregnant<br>Non-Pregnant vs Pregnant	 <br>x^2 = 8.5176<br><br><br>Z = -2.873<br>Z = -1.851<br>Z = 0.983	 <br>2	 <br>0.01414 *<br><br><br>0.01219522 *<br>0.19246150<br>0.97740244
**Sleep Minutes by Phase**<br><br>Kruskal-Wallis<br><br>Wilcox Test	 <br>x^2 = 80.063<br><br>W = 14068	 <br>1	 <br>< 2.2e-16 ***<br><br>< 2.2e-16 ***
**Day Sleep Minutes by Group Kruskal Wallis Test**	x^2 = 2.6506	2	0.2657
**Night Sleep Minutes by Group**<br><br>Kruskal-Wallis<br><br>Dunn Test<br>Male - Non-Pregnant<br>Male - Pregnant<br>Non-Pregnant - Pregnant	 <br>x^2 = 10.699<br><br><br>Z = -3.26<br>Z = -1.82<br>Z = 1.389	 <br>2	 <br>0.003342264 **<br><br><br>0.003342264 **<br>0.204006072<br>0.494620500
**Sleep Minutes Male vs Female**<br><br>Kruskal-Wallis<br><br>Wilcox Test	 <br>x^2 = 7.552<br><br>W = 2502	 <br>1	 <br>0.005994 **<br><br>0.006039 **
**Beam Counts Male vs Female**	F = 2.292	1	0.133

Journal of Experimental Biology • Supplementary information

One-way ANOVA Test	Residuals: df 129, sum sq 49.65, mean sq 0.3849		
**Period** ANOVA	F = 0.967	2	0.384
**Power**<br><br>ANOVA<br><br>Pairwise T-tests<br>Male vs Non-pregnant<br>Male vs Pregnant<br>Non-pregnant vs Pregnant	 <br>F=4.8<br><br><br>t = 2.8044, SEOD = 0.016<br>t = 2.2499, SEOD = 0.018<br>t = 0.5336, SEOD = 0.013	 <br>2<br><br><br>73	 <br>0.01010<br><br><br>0.0065 **<br>0.0275 *<br>0.5954

Table S2.

**Figure 3 Statistics**<br>Male, *n* = 28<br>Non-pregnant, *n* = 33<br>Pregnant, *n* = 31	***Variance***	***df***	***P* value**
**Sunset, Close Monitor, by Sex group**<br><br>Kruskal-Wallis<br><br>Dunn Test<br><br>Male vs Non-pregnant<br>Pregnant vs Male<br>Pregnant vs Non-pregnant	 <br>x^2 = 6.1981<br><br><br><br>Z = 1.228<br>Z = 2.487<br>Z = 1.340	 <br>2	 <br>0.04509 *<br><br><br><br>0.65806883<br>0.03859808 *<br>0.54068719
**ANOVA, Sunset, Far Monitor, by Sex group**	F = 1.819<br>Residuals: df 88, sum sq 97.56, mean sq 1.109	2	0.168
**Sunset, Both Monitors, by Sex group**<br><br>ANOVA<br><br>Pairwise T-tests<br>Male vs Non-pregnant<br>Pregnant vs Male<br>Pregnant vs Non-pregnant	 <br>F = 3.538	 <br>2	 <br>0.0311 *<br><br><br>0.19<br>0.03 *<br>1.00
**ANOVA, Sunset, by Monitor**	F = 33.33		

Close vs Far	Residuals: df 180, sum sq 213.76, mean sq 1.19	1	3.34e-08 ***
**ANOVA, Night, by Monitor**<br>Close vs Far	F = 34.89<br>Residuals: df 180, sum sq 260.5, mean sq 1.45	1	1.71e-08 ***
**ANOVA, Sunset, by Sex**<br>Male vs Female (all)	F = 6.428<br>Residuals: df 180, sum sq 244.62, mean sq 1.359	1	0.0121 *
**ANOVA, Night, by Sex**<br>Male vs Female (all)	F = 7.11<br>Residuals: df 180, sum sq 299.22, mean sq 1.662	1	0.00836 **

Table S3.

**Figure 4 Statistics**<br>Duration, Males, *n* = 100<br>Duration, Non-pregnant Females, *n* = 29<br>Duration, Pregnant Females, *n* = 20<br>Depth, Males, *n* = 100<br>Depth, Non-pregnant Females, *n* = 29<br>Depth, Pregnant Females, *n* = 20<br>Number, Males, *n* = 22<br>Number, Non-pregnant Females, *n* = 21<br>Number, Pregnant Females, *n* = 19	***Variance***	***df***	***P* value**
**Depth**<br><br>Kruskal-Wallis<br><br>Dunn<br>Male vs Non-pregnant<br>Male vs Pregnant<br>Non-pregnant vs Pregnant	 <br>x^2 = 29.461<br><br><br>Z = - 4.014<br>Z = 2.777<br>Z = 5.279	 <br>2	 <br>4.004e-07 ***<br><br><br>1.794426e-04 ***<br>1.645838e-02 ***<br>3.889976e-07 ***
**Duration**<br><br>ANOVA<br><br>Pairwise T-tests<br>Male vs Non-pregnant<br>Male vs Pregnant<br>Non-pregnant vs Pregnant	 <br>F = 3.386<br><br>Residuals: df 148, sum sq 294.90, mean sq 1.993	 <br>2	 <br><br><br><br>0.039 *<br>0.740<br>1.000
**Number**<br><br>Kruskal-Wallis<br><br>Dunn<br>Male vs Non-pregnant	 <br>x^2 = 34.22<br><br><br>Z = 2.7770	 <br>2	 <br>3.708e-08 ***<br><br><br>1.682807e-02 ***
Male vs Pregnant<br>Non-pregnant vs Pregnant	Z = 5.850<br>Z = 3.118		1.476510e-08 ***<br>5.471659e-03 ***

Journal of Experimental Biology • Supplementary information

Table S4.

**Figure 5 Statistics**<br>All groups, Expression, *n* = 6-8<br>All groups, Duration, *n* = 12<br>All groups, Progeny, *n* = 12	***Variance***	***df***	***P* value**
**Duration**			
ANOVA	F = 21.09	2	1.28e-08 ***
Pairwise T-tests	Residuals: df 44, sum sq 2409, mean sq 54.8		
Control vs Four times			3.5 e -8 ***
Control vs Four times (control)			1.000
Control vs Two times			0.0326 *
Four times vs Four times (control)			3.6 e -7 ***
Four times vs Two times			0.0006 ***
Four times (control) vs Two times			0.1835
**Expression**			
ANOVA	F = 8.647	3	
Pairwise T-tests	Residuals: df 20, sum sq 1.576, mean sq 0.0788		
Control vs Four times			0.00059 ***
Control vs Four times (control)			0.87749
Control vs Two times			0.03514 *
Four times vs Four times (control)			0.02005 *
Four times vs Two times			0.56465
Four times (control) vs Two times			0.78978
**Progeny**			
ANOVA	F = 0.071	3	
Pairwise T-tests	Residuals: df 44, sum sq 241.83, mean sq 5.496		
Control vs Four times			1.000
Control vs Four times (control)			1.000
Control vs Two times			1.000
Four times vs Four times (control)			1.000
Four times vs Two times			1.000
Four times (control) vs Two times			1.000

Journal of Experimental Biology • Supplementary information
